# A phase II trial of preoperative chemotherapy with epirubicin, cisplatin and capecitabine for patients with localised gastro-oesophageal junctional adenocarcinoma

**DOI:** 10.1038/sj.bjc.6605070

**Published:** 2009-05-12

**Authors:** N Starling, A Okines, D Cunningham, W Allum, A Wotherspoon, M Benson, J Thompson, J Thomas, G Brown, A Riddell, F Stavridi, S Ashley, J Oates, I Chau

**Affiliations:** 1 Department of Medicine, Royal Marsden Hospital NHS Foundation Trust, Surrey and London, UK; 2 Department of Surgery, Royal Marsden Hospital NHS Foundation Trust, Surrey and London, UK; 3 Department of Histopathology, Royal Marsden Hospital NHS Foundation Trust, Surrey and London, UK; 4 Department of Radiology, Royal Marsden Hospital NHS Foundation Trust, Surrey and London, UK; 5 Department of Computing and Statistics, Royal Marsden Hospital NHS Foundation Trust, Surrey and London, UK

**Keywords:** oesophageal adenocarcinoma, preoperative chemotherapy, pathological complete response

## Abstract

Preoperative cisplatin/fluorouracil is used for the treatment of localised oesophageal carcinoma. This phase II study aimed to assess the efficacy and safety of administering preoperative epirubicin/cisplatin/capecitabine (ECX). Patients with stage II or III oesophageal/gastro-oesophageal junctional adenocarcinoma from one institution received 4 cycles of ECX (epirubicin 50 mg m^−2^ day 1, cisplatin 60 mg m^−2^ day 1, capecitabine 625 mg m^−2^ b.i.d. daily) followed by surgery. The primary end point was the pathological complete response (pCR) rate based on a Simon two-stage design. Secondary end points included overall and progression-free survival (OS/PFS). Thirty-four patients were recruited: median age 60 years (range 41–81), 91% male, 97% PS 0/1, 80% T3, 68% N1. Thirty-one patients completed four ECX cycles. Grade 3/4 toxicities ⩾5% included neutropenia (62%), hand–foot syndrome (15%) and nausea/vomiting (9%). Thirteen out of 28 (46%) evaluable patients responded to chemotherapy by EUS (⩾30% reduction in maximal tumour thickness). Twenty-six out of 34 (76%) patients underwent resection (R0=73%, R1=27%). Post-operatively, two patients died within 60 days of surgery. The pCR rate was 5.9% (95% CI 0–14%) in the intent-to-treat population. According to the statistical design, this prompted early study termination. However, with a median follow-up of 34 months the median OS and 1- and 2-year survival rates were 17 months, 67 and 39% respectively. Median PFS was 13 months. Of the 14 relapsed patients, 10 presented with distant metastases. Preoperative ECX is feasible and well tolerated. Although associated with a low pCR rate, survival with ECX was comparable with published studies suggesting that pCR may not correlate with satisfactory outcome from preoperative chemotherapy for localised oesophageal adenocarcinoma.

Although the prevalence of distal gastric cancer has declined, the world-wide incidence of distal oesophageal and gastro-oesophageal junctional (GOJ) adenocarcinoma is increasing (Blot *et al*, 1991; McCann, 1999; Pera *et al*, 2005). It is estimated that over 462 000 new cases of oesophageal cancer were diagnosed world-wide in 2002 accounting for 386 000 deaths (Parkin *et al*, 2005). For patients who present with localised oesophageal adenocarcinoma surgery remains the cornerstone of treatment, potentially improving long-term survival. However, despite radical resection, 5-year survival rates range from only 15–39% (Malthaner *et al*, 2006).

Several surgical adjuncts have been investigated to improve prognosis. Preoperative chemotherapy offers the potential for tumour downstaging, early symptom improvement, enhanced resectability, the demonstration of *in vivo* chemosensitivity, the early treatment of micrometastatic disease and improvement in disease-free and overall survival (OS). This approach is supported by the results of the Medical Research Council (MRC) randomised trial of 802 patients with localised oesophageal cancer, which showed a significant improvement in 2-year survival for preoperative cisplatin/fluorouracil compared with the surgery alone (43 *vs* 34%, respectively) with both the adenocarcinoma and squamous carcinoma subgroups appearing to benefit (MRC, 2002). With a longer median follow-up of 6 years, the 5-year survival for the preoperative chemotherapy *vs* surgery alone arms was still significantly better (23 *vs* 17%) (Allum *et al*, 2008). In contrast, the US Intergroup 113 trial of 440 patients failed to show an advantage for peri-operative cisplatin/fluorouracil *vs* surgery alone in oesophageal cancer (2-year survivals of 35 *vs* 37%, respectively) leading to variation in the uptake of this practice (Kelsen *et al*, 1998). However, in a separately published subgroup analysis, responders to preoperative chemotherapy (as gauged by barium oesophagrams) had a significantly better outcome than non-responders (Kelsen *et al*, 2007). In a systematic review of 11 randomised studies involving 2019 patients with oesophageal carcinoma, preoperative cisplatin-based chemotherapy appeared to confer a survival advantage with a hazard ratio (HR) of 0.88 (95% confidence interval (CI) 0.75–1.04) (Malthaner *et al*, 2006).

The preoperative chemotherapy approach to localised oesophageal cancer may be optimised by the incorporation of more effective cytotoxic agents. The epirubicin/cisplatin/fluorouracil (ECF) triplet regimen is effective in the treatment of advanced gastro-oesophageal cancer (Webb *et al*, 1997; Ross *et al*, 2002) and when given as part of a peri-operative strategy for the treatment of localised gastric and lower oesophageal/GOJ adenocarcinoma, resulting in a significant improvement in 5-year survival compared with surgery alone (36 *vs* 23%) (Cunningham *et al*, 2006). Recently, capecitabine (X) and oxaliplatin (O) were found to be as effective as fluorouracil and cisplatin, respectively, when substituted in the ECF regimen for treating advanced gastro-oesophageal cancer (Cunningham *et al*, 2008). The epirubicin/cisplatin/capecitabine (ECX) and EOX regimens provide more convenient treatment alternatives to ECF (no requirement for an indwelling central venous access device or hydration for oxaliplatin) with certain toxicity advantages.

In patients with oesophageal adenocarcinoma long-term survival is strongly correlated with the achievement of pathological complete response (pCR) to neoadjuvant chemoradiotherapy (Walsh *et al*, 1996; Geh *et al*, 2001; Urba *et al*, 2001; Berger *et al*, 2005; Burmeister *et al*, 2005; Rohatgi *et al*, 2005). However, in the US Intergroup and OEO2 trials of preoperative chemotherapy, the pCR rates were only 2 and 4%, respectively (Kelsen *et al*, 1998; MRC, 2002). With the emergence of more effective combination chemotherapy regimens there is potential to improve preoperative chemotherapy-associated pCR rates and thus survival. As a potential surrogate for outcome, pCR could facilitate the reporting of results from phase II trials, OS being the preferred outcome measure for phase III trials.

The aim of this single centre phase II study was to assess the pCR rate associated with preoperative ECX in patients with localised, operable oesophageal and GOJ adenocarcinoma and to evaluate OS, progression-free survival (PFS), toxicity and patterns of treatment failure.

## Methods

### Patients

Eligible patients were older than 18 years, had histologically proven adenocarcinoma of the thoracic oesophagus or GOJ (Siewert's types I–III), American Joint Committee on Cancer (AJCC) stage II or III (T2-3, N0-1, M0) disease, an Eastern Cooperative Group (ECOG) performance status ⩽2 and adequate renal, hepatic and bone marrow function. Exclusion criteria included locally advanced (T4) or metastatic disease (including M1a), prior chemotherapy or radiotherapy, any clinically significant uncontrolled co-existing illness or previous malignant disease and complete dysphagia. This single institution trial was approved by the local Ethics and Scientific Review Committees and all patients provided written informed consent.

### Pre-treatment evaluation

Patients underwent staging computed tomography (CT) of the chest/abdomen/pelvis and endoscopic ultrasound (EUS) within 28 days of the first treatment. Laparoscopy and positron emission tomography (PET) were performed where indicated. Glomerular filtration rate was determined by 24 h urinary clearance or EDTA testing. Audiography and echocardiography or multiple-gated acquisition scanning were performed when clinically indicated. Operability was determined by a multi-disciplinary team following this evaluation.

### Treatment

#### Preoperative chemotherapy

Chemotherapy was administered for four cycles preoperatively. Each cycle comprised epirubicin (50 mg m^−2^) on day 1 by intravenous bolus, cisplatin (60 mg m^−2^) intravenously on day 1 with pre- and post-hydration and oral capecitabine (625 mg m^−2^ twice daily) continuously throughout treatment. Before each cycle of chemotherapy, a full blood count and biochemical function (including renal and liver parameters) were checked. Adverse events were assessed at every treatment visit and were graded according to the National Cancer Institute Common Toxicity Criteria (NCI-CTC) version 2.0. Dose modifications were instituted as previously described (Sumpter *et al*, 2005). Cisplatin was discontinued in the presence of clinically significant ototoxicity or peripheral neuropathy and substituted by carboplatin at the discretion of the investigator. Epirubicin was omitted if there was a clinically significant deterioration in cardiac function confirmed by echocardiography (although cumulative dose only 200 mg m^−2^).

#### Surgery

Post-chemotherapy evaluation included repeat CT chest/abdomen/pelvis and EUS reviewed by the multi-disciplinary team. Patients with stable or responding disease who remained fit for surgery proceeded to surgery 4–6 weeks after the last cycle of chemotherapy. Surgery was undertaken within one regional high-volume centre (the majority by one surgeon) and was determined by the location and extent of the localised tumour. Patients with Siewert type I or II junctional cancers were treated by right thoraco-abdominal oesophago-gastrectomy with two field lymph node dissection. Those with type III tumours underwent extended total gastrectomy with D2 lymphadenectomy using either a transhiatal approach or a left thoraco-abdominal approach. Resection of other organs was only included if there was evidence of local infiltration. Post-operative complications, and any deaths following surgery were recorded.

Patients deemed inoperable due to progression with loco-regional disease were considered for definitive chemoradiation where appropriate. Patients deemed inoperable at surgery due to metastatic disease were followed up and treated according to the local protocols.

### Post-treatment evaluation and follow-up

Resected tumours were reviewed for histopathological response by one histopathologist according to TMN staging after neoadjuvant chemotherapy. The specimens were inflated and pinned to reduce shrinkage of the oesophagus, sliced perpendicular to the lumen in their entirety to give 5 mm slices and each slice was embedded and processed for histological examination. The whole length of the tumour was examined to exclude the possibility of missing small foci of residual tumour. Tumours were examined for pCR defined as the inability of the pathologist to identify viable malignant cells within the resected specimen. All patients had a post-surgery baseline CT scan and were followed up at 3 monthly intervals for the first year, 6 monthly intervals for the second year and annually thereafter. CT scans (chest/abdomen/pelvis) were routinely performed at 1 and 2 years after surgery and otherwise when clinically indicated.

### Statistical analysis

The primary end point was the pCR rate in the intent-to-treat population. A Simon's two-stage design (Chen and Shan, 2008) was used based on a lower limit of a pCR rate of 5% (p_0_) and an acceptable pCR rate of 15% (p_1_). With a calculated sample size of 33 patients, the study would stop early if a response rate of lower than 5% was observed with 80.1% power (*α*=0.049). Thus, if ⩽2 pCRs were observed, the study would terminate due to lack of efficacy, conversely if ⩾6 pCRs were observed, then a conclusion of efficacy would be reached and the trial stopped. If neither criteria was met, the study would proceed to the second stage, recruiting an additional 20 patients (total *n*=53), concluding efficacy only if ⩾6 pCRs were observed. Seven additional patients could be recruited to address the potential dropouts.

Secondary end points included OS and PFS, objective response rate by CT and EUS (EUS response was considered to be >30% reduction in maximal tumour thickness), toxicity and patterns of treatment failure. Overall survival was calculated from the date of study registration to the date of death from any cause. Progression-free survival was calculated from the date of study registration to the date of first documented progression or death. Patients remaining alive or lost to follow-up were censored at the date of last follow-up. Survival was calculated according to the Kaplan–Meier method.

## Results

### Patients

Between November 2002 and June 2007 34 patients were recruited, all eligible for the study. The median age was 60 years (range 41–81 years), most patients were of performance status 0/1 and the majority had T3 disease (79.4%) with nodal involvement as assessed by EUS. Patient characteristics are indicated in Table 1.

### Treatment

#### Chemotherapy

The median number of preoperative cycles of chemotherapy delivered was 4. Thirty-one patients completed all four cycles. Two patients received only 1 and 3 cycles, respectively, due to progressive disease on therapy as assessed by CT, and one patient received three cycles due to toxicity. For the cohort of 34 patients, the mean cumulative doses of epirubicin, cisplatin and capecitabine were 181, 221 and 93 g m^−2^, respectively (i.e., 90, 92 and 89% of the planned cumulative doses, respectively). By EUS, 13 of the 28 assessable patients had a response and 15 patients had stable disease.

#### Surgery

Twenty-six patients were resected (76% of the 34 patients) after preoperative chemotherapy. One additional resected patient did not have protocol-specified preoperative therapy and was excluded from the resection analysis (considered inoperable after four cycles of chemotherapy, but resected after a subsequent course of chemoradiation). Reasons for not proceeding to resection included progressive disease during preoperative chemotherapy (*n*=2), inoperable at laparotomy due to peritoneal/liver metastases undetected by preoperative imaging (*n*=4) and tumour considered too bulky on preoperative imaging (*n*=1). Of the 26 resected patients, 21 patients underwent oesophago-gastrectomy and 6 patients underwent extended total gastrectomy. Nineteen patients achieved R0 resections (no evidence of residual macroscopic or microscopic disease at margins) (73%; 95% CI 56−90 or 56% on an intent-to-treat basis) and seven had R1 resections by virtue of microscopic disease within 1 mm of the circumferential resection margins. Of the seven patients who had R1 resections, four received radical post-operative chemoradiotherapy.

### Pathological findings

In the group of 26 resected patients, pCR was seen in 2 patients which represents a pCR rate of 7.4% (95% CI 0–17) or 5.9% (95% CI 0–14) in the intent-to-treat population, which met the criteria for early study termination due to lack of treatment efficacy based on pCR. Twelve patients experienced pathological T downstaging compared with the initial EUS T stage and five of these were EUS responders.

### Survival

**Figure 1:**
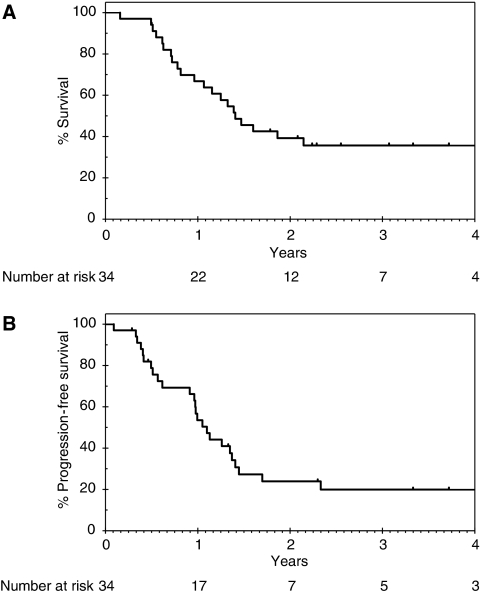
Overall and progression-free survival in the intent-to-treat population. (**A**) Overall survival. (**B**) progression-free survival.

**Figure 2:**
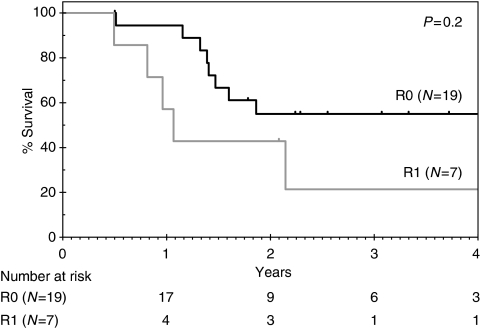
Overall survival according to R resection status (*N*=26).

**Figure 3:**
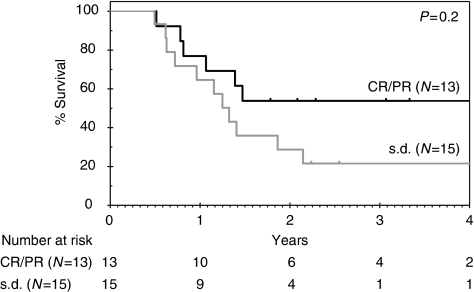
Overall survival according to EUS response (*N*=28) – on the basis of evaluable patients for whom measurement of maximal tumour thickness was available pre- and post-chemotherapy. Reasons for patients being non-evaluable included death from progressive disease (*n*=1), stenotic lesion (*n*=1), EUS data unavailable (*n*=2), thickness not recorded on second EUS (*n*=2).

With a median follow-up of 34 months, 12 patients remain alive (9 disease-free). For the 34 patients in the intent-to-treat population, the median OS is 17 months (95% CI 12–22) and 1 and 2-year survivals are 67% (95% CI 48–80%) and 39% (95% CI 23–55%), respectively (Figure 1A). The median PFS is 13 months (95% CI 11–16), Figure 1B. Overall survival according to R resection status for the 26 resected patients is shown in Figure 2 and indicates a nonsignificant trend for improved survival in the R0 *vs* R1 group (HR 2.1; 95% CI 0.7–7.0; *P*=0.2). Both patients who achieved pCR developed systemic recurrent disease within 1 year (one with brain metastases and the other with peritoneal disease). In an exploratory analysis, OS according to EUS response was performed (Figure 3) and indicates a nonsignificant trend for improved survival for EUS responders compared with non-responders (HR 2.0; 95% CI 0.9–5.1; *P*=0.2). Eleven patients proceeded to receive systemic chemotherapy for advanced disease.

### Chemotherapy-related toxicity and surgical complications

Adverse events that occurred during chemotherapy are documented in Table 2. There were no chemotherapy-related deaths during preoperative chemotherapy. One patient who received only one cycle of chemotherapy died within 30 days of chemotherapy of objectively documented progressive disease. One patient died after 54 days of surgery with a chronic history of chest problems complicated by surgery and one other died after 31 days due to post-operative chylous leak complicated by renal failure (malignant cells were identified in the chylous fluid). Post-operative complications occurring in greater than 5% of patients included respiratory complications (*n*=5), chylous leak (*n*=2), recurrent laryngeal nerve palsy (*n*=2) and renal failure (*n*=2). For the 26 patients undergoing resection, first sites of recurrence are indicated in Table 3.

## Discussion

In this phase II study of 34 patients with operable gastro-oesophageal adenocarcinoma, preoperative ECX was associated with a pCR rate of only 5.9% in the intent-to-treat population. This resulted in early termination of the study based on the statistical premise of the trial, which used pCR as the primary end point. This raises doubts over whether more effective chemotherapy regimens lead to improved pCR rates compared with those observed in trials of preoperative cisplatin/5FU (Kelsen *et al*, 1998; MRC, 2002) and highlights the potential limitations of pCR as a primary end point and surrogate marker of outcome for preoperative chemotherapy. Paradoxically, both patients who achieved pCRs in this study relapsed within 1 year of surgery. The early treatment and potential elimination of micro-metastatic disease may actually be the critical factor in conferring a survival advantage to systemic neo-adjuvant therapy for localised oesophageal cancer. Alternative regimens, for instance those incorporating docetaxel, may result in higher pCR rates in phase II evaluation (Lorenzen *et al*, 2007) but whether this translates into improved survival has yet to be shown. The median and 2 year survivals of 17 months and 39%, respectively, are comparable to those observed in the MRC and Intergroup trials of preoperative cispatin/fluorouracil (Kelsen *et al*, 1998; MRC, 2002). However, our study was not powered around survival, included patients mainly with T3 N1 disease and excluded squamous carcinoma, which may account for the reason for survival that did not appear to be greater with an effective triplet regimen. Another consideration is the accuracy of histological assessment of pCR, particularly when used as a primary end point. However, standard histopathological review protocols are likely to be accurate in this regard (Chang *et al*, 2007), and in our institution all histopathological examination was performed by a single experienced gastrointestinal pathologist. There are no other validated scores of tumour regression in response to preoperative therapy in oesophageal cancer, although recently one group defined responders and non-responders as those with ⩽ or >10% residual tumour cells, respectively, in the resected specimen (Weber *et al*, 2001; Brucher *et al*, 2006; Ott *et al*, 2006), and thus pCR remains the standard assessment of histopathological response.

The PCR rates associated with preoperative chemoradiation, the other major treatment strategy for localised oesophageal carcinoma commonly used in North America, range from 15 to 33% for the randomised controlled trials, which included patients with adenocarcinoma (Walsh *et al*, 1996; Urba *et al*, 2001; Burmeister *et al*, 2005; Tepper *et al*, 2008). Although higher than those achieved with preoperative chemotherapy, these pCR rates have failed to translate into a consistent survival advantage for preoperative chemoradiation, the two positive studies being criticised for unusually poor survival in the surgery alone arm (Walsh *et al*, 1996) and for early termination due to poor recruitment (Tepper *et al*, 2008). A recent meta-analysis of trials of preoperative therapy in localised oesophageal carcinoma showed a similar survival advantage for the subgroup of patients with adenocarcinoma treated with chemoradiation (*n*=345, HR 0.75, 95% CI 0.59–0.95; *P*=0.02) and chemotherapy alone (*n*=533, HR 0.78, 95% CI 0.64–0.95; *P*=0.014) (Gebski *et al*, 2007). Notably, there appeared to be a survival advantage in the pooled analysis for squamous carcinoma treated with preoperative chemoradiation but not preoperative chemotherapy, in contrast to observations from subgroup analysis by histology of the MRC trial (Allum *et al*, 2008). Differential treatment effects have earlier been noted according to histology underscoring that squamous carcinoma and adenocarcinoma are separate clinical and biological entities.

The toxicity associated with preoperative ECX was reasonably consistent with that reported for the regimen in the treatment of advanced disease (Cunningham *et al*, 2008). The R0 resection rate of 73% is higher than that reported in the MRC and Intergroup trials (60 and 63%, respectively) (MRC, 2002; Kelsen *et al*, 1998) and the frequency of post-operative complications was low. Subgroup analysis in both the MRC and Intergroup trials indicated that R0 resection was associated with significantly better survival as compared with R1 resection or less with a 3-year OS of 42 *vs* 18% (Allum *et al*, 2008) and 39 *vs* 12% (Kelsen *et al*, 2007) for R0 *vs* R1 resection, respectively. In this study, there was a trend towards improved survival for patients achieving an R0 compared with an R1 resection, which did not reach statistical significance and is likely to reflect the small patient numbers. The majority of patients relapsing after resection presented with distant metastases, consistent with earlier observations in oesophageal and GOJ adenocarcinoma (Wayman *et al*, 2002), and highlighting the need for surgical adjuncts incorporating effective systemically directed therapies in this disease. Preoperative approaches remain the most extensively investigated with only a limited number of small negative randomised trials assessing adjuvant therapy mainly in squamous carcinoma (Malthaner *et al*, 2004).

Accurate assessment of response to preoperative therapy and its correlation with outcomes may facilitate the rationalisation of further treatment, limit surgery-related morbidity and mortality and optimise outcomes. As compared with CT and oesophago-gastroscopy, EUS is more reliable for determining response to preoperative therapy (Brown *et al*, 2004; Westerterp *et al*, 2005). In an exploratory analysis, we attempted to assess the potential of EUS as a tool for outcome prediction to preoperative chemotherapy, evaluating the change in maximal tumour thickness with a decrease of ⩾30% representing a response, the cut-off value utilised in RECIST and a simple response classifier. Response according to EUS and its correlation with outcomes, such as pathological response and survival, has previously been shown in small studies of neoadjuvant therapy for oesophageal cancer (Hirata *et al*, 1997; Chak *et al*, 2000; Willis *et al*, 2002; Masaho *et al*, 2005). In our analysis, there was no difference in OS between EUS responders and non-responders to preoperative chemotherapy and is likely to reflect the small number of patients. In addition, EUS is limited in its inability to distinguish post-treatment fibrosis and inflammation from tumour (Lightdale and Kulkarni, 2005). A more promising imaging biomarker for outcome prediction to preoperative chemotherapy further along in evaluation is metabolic response by PET. Having been identified retrospectively (Weber *et al*, 2001) and validated prospectively (Ott *et al*, 2006), the feasibility of an early metabolic response-adjusted treatment algorithm has been confirmed in a non-randomised trial (Lordick *et al*, 2007), and randomised assessment of this biomarker-driven treatment strategy is planned. There is also currently extensive investigation to identify predictive and prognostic molecular markers and signatures.

Efforts to improve the preoperative chemotherapy approach to localised oesophageal cancer include the incorporation of newer more effective cytotoxics including the taxanes, oxaliplatin and capecitabine and targeted agents such as bevacizumab. In an ongoing UK phase III trial of the MRC patients with oesophageal/GOJ (types I and II) adenocarcinoma are randomised between two cycles of cisplatin/fluorouracil and four cycles of ECX with OS as the primary end point. This study was designed as a prelude to the MRC trial, partly to provide some initial safety data on preoperative ECX, as the randomised study of ECF *vs* the capecitabine and oxaliplatin regimens in the advanced disease setting (Cunningham *et al*, 2008) had not reported at that time. Many patients at our institution preferentially enrolled in the randomised trial following its launch in 2004. In another ongoing trial of the MRC, patients with gastric and type III GOJ adenocarcinoma are randomised between six cycles of pre- and post-operative ECX or the same with the addition of bevacizumab. The present phase II trial indicated the safety and feasibility of administering preoperative ECX for oesophageal and GOJ tumours, that preoperative chemotherapy is associated with low pCR rates and the limitations of pCR as a surrogate marker of outcome for preoperative chemotherapy.

## Figures and Tables

**Table 1:** Patient characteristics

	***N*=34**
Median age, years (range)	60 (41–81)
	
*Sex*	
Male	31 (91%)
Female	3 (9%)
	
*ECOG performance status*	
0	9 (26%)
1	24 (71%)
2	1 (3%)
	
*Histology*	
Adenocarcinoma	34 (100%)
	
*Siewert's classification*	
Type I	18 (53%)
Type II	12 (35%)
Type III	4 (12%)
	
*T stage*	
T2	7 (21%)
T3	27 (79%)
	
*N Stage*	
N0	11 (32%)
N1	23 (68%)
N2	0 (0%)
	
*M stage*	
M0	34 (100%)

**Table 2:** Adverse events during preoperative chemotherapy (*N*=34)

	**All grades, *n* (%)**	**Grade 3/4, *n* (%)**
*Haematological*		
Neutropenia	32 (94)	21 (62)
Anaemia	26 (76)	3 (9)
Thrombocytopenia	8 (24)	0 (0)
Febrile neutropenia	1 (3)	1 (3)
		
*Gastrointestinal*		
Nausea and vomiting	26 (76)	3 (9)
Diarrhoea	15 (44)	1 (3)
Stomatitis	13 (38)	0 (0)
		
*Skin*		
Hand–foot syndrome	22 (64)	5 (15)
		
*Neurological*		
Peripheral neuropathy	8 (24)	1 (3)
		
*Constitutional*		
Fatigue	34 (100)	1 (3)

**Table 3:** First sites of recurrence for patients undergoing resection (*N*=26) (documented relapse, *N*=14)

	**Number of patients**	
**Site of recurrence**	**R0, *N*=19**	**R1, *N*=7**
Local – (resection bed/anastomosis/ local lymph nodes)	1	3
Distant^a^	5	3
Local and distant	2	0
No recurrence	9	0
Death without documented recurrence	2	1

a Four patients showed recurrence at more than one site. Sites of distant recurrence included distant nodes (*N*=6), peritoneum (*N*=3), lung (*N*=2), brain (*N*=1), liver (*N*=1) and bone (*N*=1).
